# Survival Outcome of Retroperitoneal Sarcomas Treated With a Surgery-First Approach: A Single-Center Experience

**DOI:** 10.7759/cureus.49818

**Published:** 2023-12-02

**Authors:** Pradeep Chandran, Joseph Francis, Alex Chakiath, Sulfekar Meera Sainaba, Prashant Girijavallabhan Nair, Jayas Siby, Gowri Madhusudanan Pillai, Jasira Padinhare Madathil, Martin Verheij

**Affiliations:** 1 Trauma Surgery, King's College Hospital, London, GBR; 2 Plastic and Reconstructive Surgery, King Edward Memorial Hospital and Seth Gordhandas Sunderdas Medical College, Mumbai, IND; 3 Surgical Oncology, Malabar Cancer Centre, Thalassery, IND; 4 General Surgery, Government Medical College, Thiruvananthapuram, IND; 5 General Surgery, Wirral University Teaching Hospital NHS Foundation Trust, Liverpool, GBR; 6 General Surgery, Whipps Cross University Hospital, London, GBR; 7 General Surgery, West Middlesex University Hospital, London, GBR; 8 Breast and General Surgery, Homerton University Hospital, London, GBR

**Keywords:** retroperitoneum, liposarcoma, rps, retroperitoneal sarcoma surgery, retroperitoneal sarcoma

## Abstract

Background

Retroperitoneal sarcomas (RPS) are rare and complex tumors originating from the retroperitoneal space, an anatomical region nestled behind the abdominal cavity and shielded by the posterior abdominal wall. Late clinical presentation is a hallmark of retroperitoneal sarcomas. The symptoms are often nonspecific, and nodal metastases are rare. Computed tomography (CT) remains the investigation of choice, and a preoperative biopsy is usually not needed. Surgical resection remains the mainstay of treatment, along with adjuvant radiation and chemotherapy. Survival rates are in general poor, even after complete resection. In this study, we attempt to shed some light on the clinicopathological profiling of retroperitoneal sarcomas and their survival outcomes.

Objective

The objective of this study is to assess the demographic, clinical, and pathological profiling of patients with retroperitoneal sarcoma and to study the survival of patients with retroperitoneal sarcoma.

Methodology

We conducted a hospital-based retrospective observational study in a tertiary care center in South India between January 2011 and January 2021. We included all patients with histopathologically proven retroperitoneal sarcoma. Metastatic cases and those who underwent chemotherapy or radiation prior to presentation were excluded. Their demographics, pathological reports, and survival were followed up and collected, and statistical analysis was done.

Results

The study included 16 cases with retroperitoneal sarcomas across the decade in which the data was collected, confirming the rarity of the tumor, out of which more than 40% of patients were above the age of 60. The most common symptom was found to be a bloating sensation in nine patients, followed by abdominal pain in three patients. Seventy-five percent of the patients were found to have a T4 (i.e., a size of more than 15 cm) tumor at presentation. Well-differentiated liposarcoma was found to be the most common pathological variant accounting for 25% of the cases. The mean survival was found to be 8.05 years, which dropped to 5.74 years in Grade 3 tumors.

Conclusion

Retroperitoneal sarcomas are rare tumors of which liposarcoma is the most common variant. A significant reduction in the mean survival was identified in Grade 3 sarcomas compared to the cumulative survival time of Grade 1 and Grade 2 retroperitoneal sarcomas.

## Introduction

Soft tissue sarcomas are a rare group of mesenchymal tumors, which account for approximately 1% of all malignant tumors [1]. Ten percent of all sarcomas occur in the retroperitoneum [1]. Some sarcomas are more common in the retroperitoneum. The most common type seen in the retroperitoneum is liposarcoma, followed by leiomyosarcoma [2]. Since the retroperitoneum lacks distinct anatomical compartments, these tumors grow into large sizes and invade adjacent structures, before becoming symptomatic [3]. Surgical resection is the mainstay of treatment, as there is little benefit from chemotherapy or radiotherapy [4]. Even with advanced imaging techniques, which help to accurately define the lesion preoperatively and thereby allow us to adopt radical resection techniques, it still remains a challenge for surgeons to get a microscopically negative resection margin [4]. These tumors, also, have a high propensity for recurrence, thereby affecting the overall survival and quality of life of these patients [4].

Sarcomas are, often, called an orphan disease, which needs to be treated at dedicated integrated treatment facilities [5]. With this study, we are aiming to report our experience with these tumors, in a tertiary care center in a developing country, with limited resources, with a surgery-first approach.

## Materials and methods

This was a retrospective analysis of a prospectively maintained database of patients who underwent retroperitoneal sarcoma (RPS) excision with curative intent at a tertiary care center in South India from the year 2011 to 2021. Patients who underwent neoadjuvant therapy prior to excision; those who were diagnosed with gastrointestinal stromal tumor (GIST), desmoids, or visceral sarcomas; and those below the age of 13 were excluded from the study. Patients with borderline performance status of Eastern Cooperative Oncology Group (ECOG) Performance Status (PS) 2 and poor performance status of ECOG PS 3 and 4 were also excluded from the study.

All patients underwent thorough evaluation including history and clinical examination. All patients were staged with the help of contrast-enhanced computed tomography (CT) imaging of the abdomen and thorax. Image-guided biopsy was taken in order to establish the histological diagnosis. Any pathology reports available from outside were reviewed by the in-house pathologists to reach a consensus on the diagnosis. The patient's case record was brought to the multidisciplinary tumor board, and the final treatment plan was made.

A wide local excision of the tumor was done. The specimen was oriented and measured. Histopathological examination was done, and the tumor was classified according to the World Health Organization classification. Tumor grade was classified using the Federation Nationale des Centres de Lutte Contre Le Cancer (FNCLCC) system, which includes tumor differentiation, necrosis, and mitotic rate per high-powered field.

Adjuvant treatment was decided on the postoperative histology, grade, tumor size, and margin status after bringing the histopathology report to the multidisciplinary board. The patients were then followed up three monthly for the first two years, six monthly for the next three years, and yearly thereafter with thorough clinical examination and chest X-ray at every visit. An imaging of the abdomen was done at the treating physicians' discretion in the presence of symptoms. Local recurrence was defined as disease recurrence noted in the retroperitoneum, and distant recurrence was defined as disease recurring anywhere else including the lung, liver, bone, and nodes.

The obtained data was analyzed using the Statistical Package for Social Sciences (SPSS) software (IBM SPSS Statistics, Armonk, NY). Descriptive statistics were described using mean, median, and percentages. The Kaplan-Meier method was used to calculate the survival. The statistical significance was defined as a p value of less than 0.05. The overall survival was defined as the time period from the date of surgical resection to the date of death or the date of last follow-up. The disease-free survival was defined as the time period from the date of surgery to the date of recurrence, when the recurrence was documented by either radiology or pathological examination. The primary end point of the study was to identify the overall survival. The secondary end point was to describe the demographic, clinical, and pathological profile of the patients with retroperitoneal sarcoma who presented to our institution.

## Results

A total of 16 patients were included in the study. The majority of them were above the age of 50 (n=13, 81.2%), with a median age of 59. Fifty percent (n=8) of them were males. Twenty-five percent of the patients have ECOG Performance Status 0. The rest of them have Performance Status 1. The most common presentation was a bloating sensation in the abdomen, which was seen in 56.25% (n=9) of the patients; 18.75% (n=3) presented with pain in the abdomen. One patient (6.25%) presented with mass per abdomen, bleeding per rectum, and hematuria.

Seventy-five percent (n=12) of the tumors were found to be more than 15 cm in size (T4), 18.8% (n=3) were found to be between 10 and 15 cm in size (T3), and the remaining one patient (6.3%) was found to have tumor between 5 and 10 cm (T2). All surgeries performed were R0 excisions. Histological subtypes and the final pathology are given in Table 1.

**Table 1:** Histological subtypes

Histology	Number	Percent
Liposarcoma	11	68.75%
Well-differentiated liposarcoma	4	25%
Myxoid liposarcoma	1	6.25%
Dedifferentiated liposarcoma	6	37.5%
Leiomyosarcoma	2	12.5%
Malignant peripheral nerve sheath tumor	2	12.5%
Malignant fibrous histiocytoma	1	6.25%

The grades of tumors are as depicted in Table 2.

**Table 2:** Grade of tumor

Grade of tumor	Number	Percentage
Grade 1	7	43.8%
Grade 2	4	25%
Grade 3	5	31.3%

Of the patients, 43.8% (n=7) did not receive any adjuvant treatment. Two (12.5%) patients among the Grade 1 tumors and Grade 2 tumors received adjuvant treatment. All patients who had a final histopathology report of Grade 3 received adjuvant treatment. Among those who received adjuvant treatment, 12.5% (n=2) received only chemotherapy, 31.3% (n=5) received adjuvant radiotherapy, and 12.5% (n=2) received adjuvant concurrent chemoradiation.

**Figure 1:**
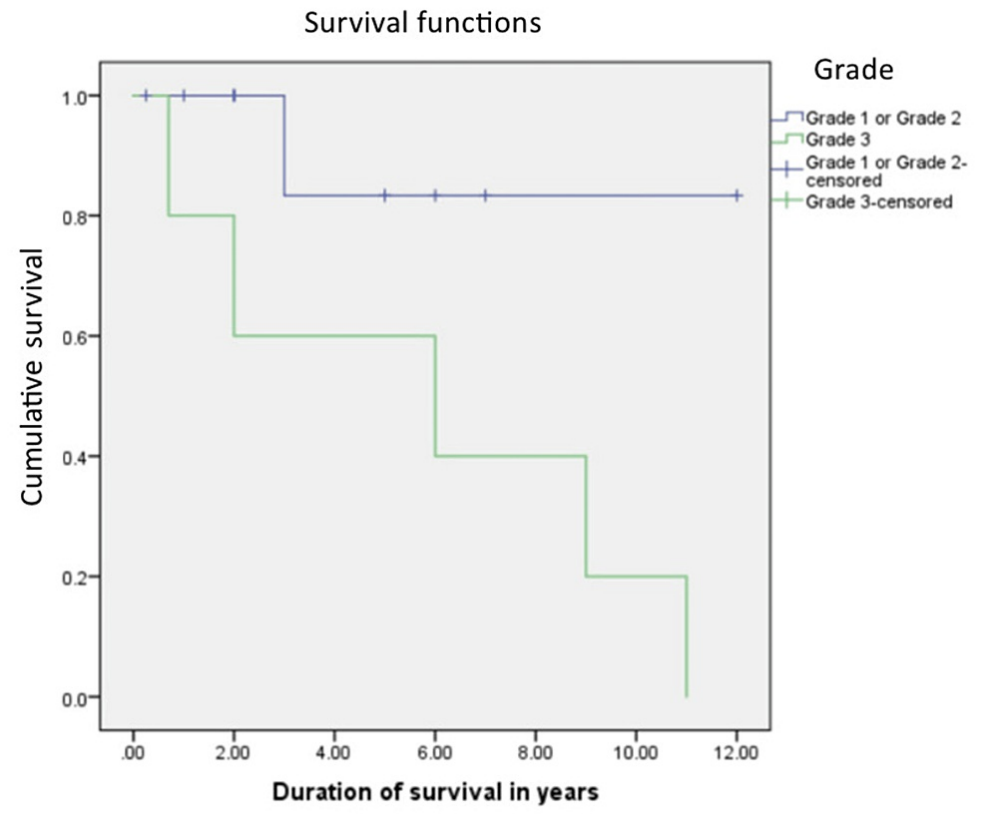
The Kaplan-Meier curve showing survival in Grade 1 or 2 tumors as compared to Grade 3 tumors

**Figure 2:**
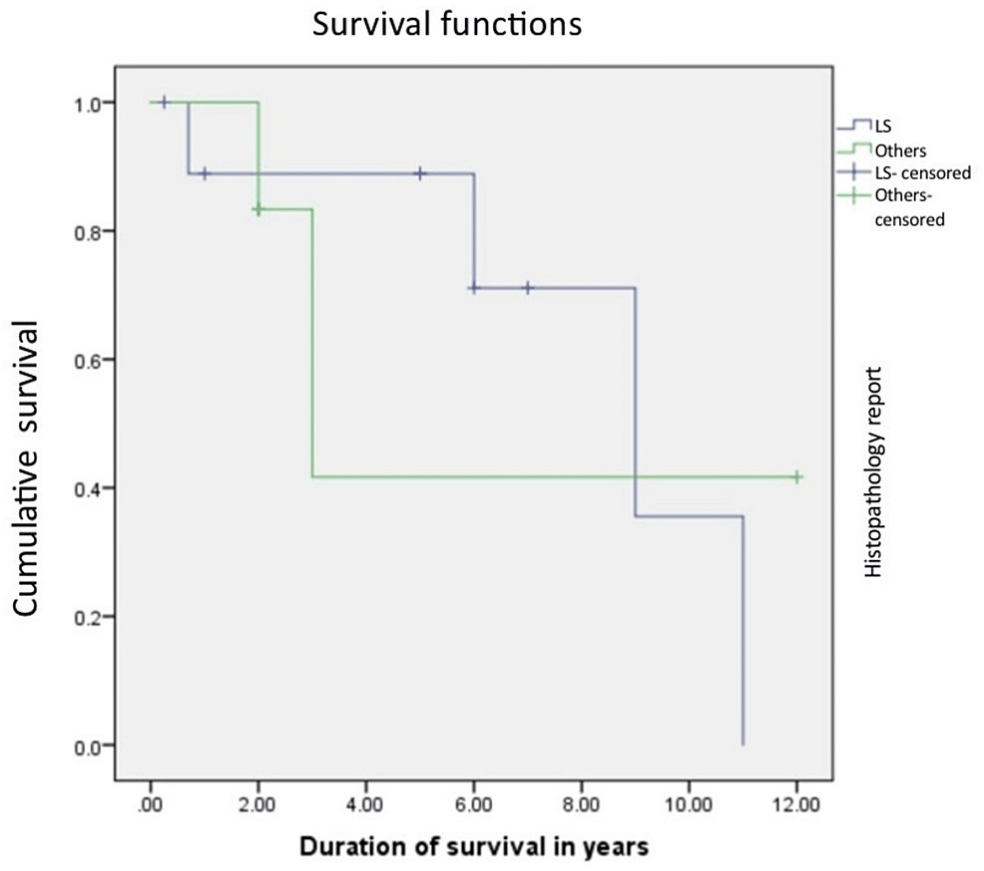
The Kaplan-Meier curve showing survival in liposarcoma (LS) compared to other histologies

**Figure 3:**
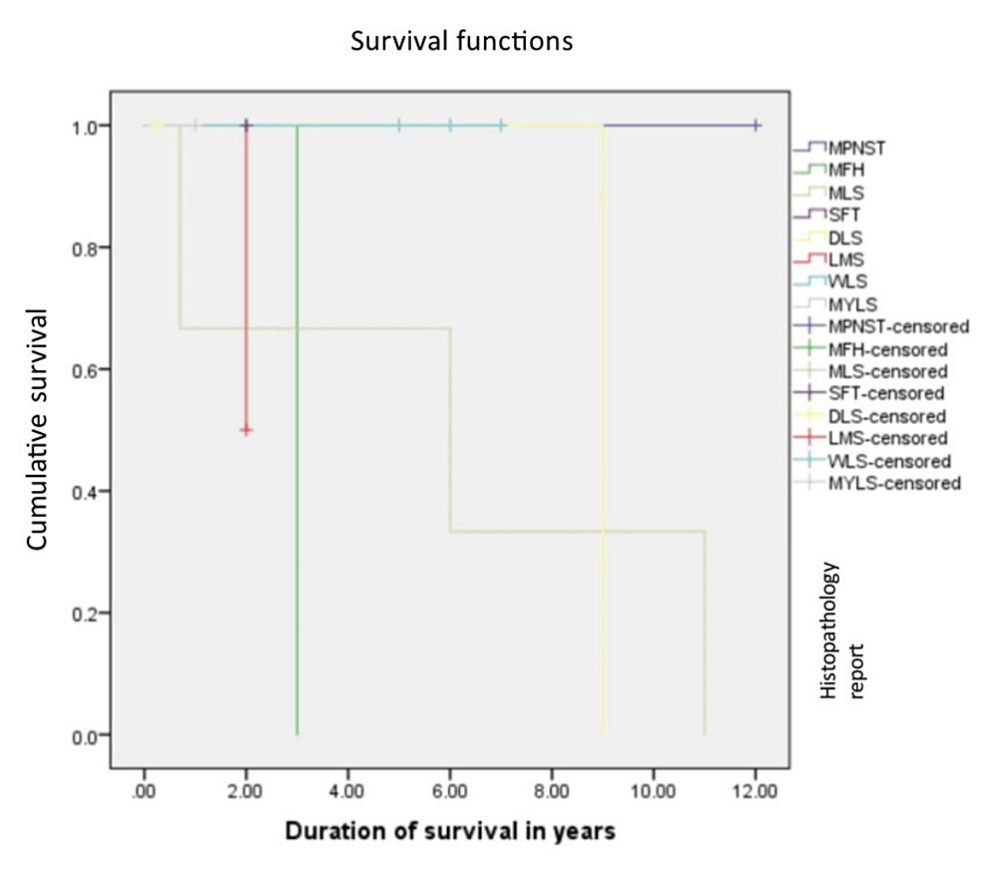
The Kaplan-Meier curve showing survival in different histological types of retroperitoneal sarcoma (RPS) MPNST, malignant peripheral nerve sheath tumor; MFH, malignant fibrous histiocytoma; MLS, mixed liposarcoma; SFT, solitary fibrous tumor; DLS, dedifferentiated liposarcoma; LMS, leiomyosarcoma; WLS, well-differentiated liposarcoma; MYLS, myxoid liposarcoma

**Figure 4:**
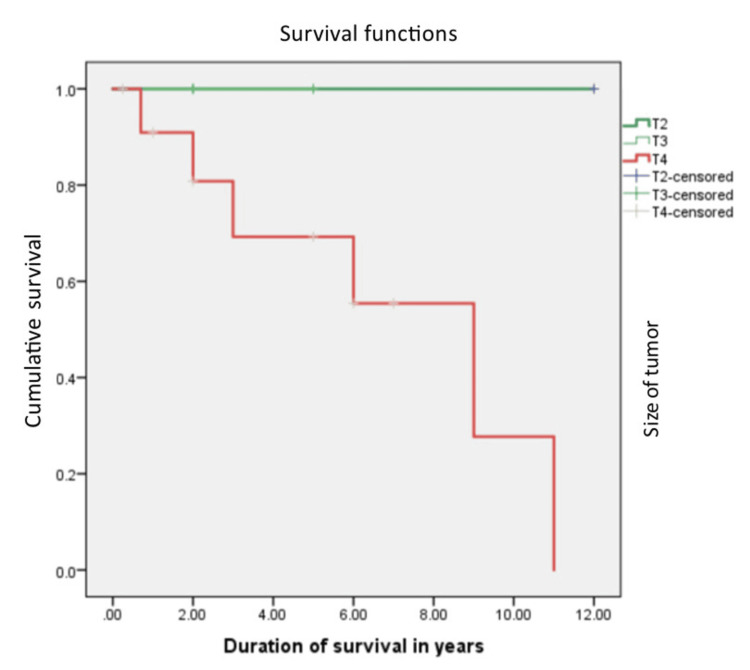
The Kaplan-Meier curve showing survival in in different sizes of tumor stages

The median overall survival was 108 months. The relationship between the grade of the tumor, the T stage of the tumor, the histopathology of the tumor, and the overall survival rate was calculated. Patients with Grade 1 and 2 tumors were found to have a better overall survival of 126 months, compared to 72 months in Grade 3 tumors, which was statistically significant with a p value of 0.045 with 95% confidence interval, and this is shown in Figure 1. Liposarcoma had a median overall survival of 108 months, while the other histologies cumulatively had a median survival of 36 months, which was not statistically significant (p=0.9), and this is shown in Figure 2. Figure 3 depicts the survival comparison of different histological varieties of retroperitoneal sarcomas. The T stage of the tumor was not significantly associated with the overall survival (p=0.2) and is shown in Figure 4. The five-year survival rate was 75%.

## Discussion

The retroperitoneum is a complex anatomical space that is bounded superiorly by the 12th rib and vertebra, inferiorly by the sacrum and the iliac crest, anteriorly by the peritoneum, posteriorly by the posterior abdominal muscles, and laterally by the lateral margins of the quadratus lumborum muscle [1]. Tumors arising here have the ability to attain a very large size, without producing any symptoms. Less commonly, when they do present with symptoms, they are often very nonspecific, such as vague abdominal pain and fullness [6]. Consequently, these symptoms are usually dismissed without proper evaluation, resulting in very large tumors at the time of diagnosis.

These tumors are most commonly reported in the sixth decade of life [6], and the median age of diagnosis in our study was found to be 59 years. Concurring with the existing evidence that incidence rates are similar in both sexes, we also noted an equal gender distribution [5].

The staging widely accepted is that of the American Joint Committee on Cancer staging (AJCC eighth edition), which stages RPS as shown in Table 3 [7].

**Table 3:** AJCC staging of retroperitoneal sarcoma AJCC: American Joint Committee on Cancer

T/N/M	Criteria
T1	Tumor = 5 cm in greatest dimension
T2	Tumor > 5 cm and = 10 cm in greatest dimension
T3	Tumor > 10 cm and = 15 cm in greatest dimension
T4	Tumor > 15 cm in greatest dimension
N0	No regional lymph node metastasis or unknown lymph node status
N1	Regional lymph node metastasis
M0	No distant metastasis
M1	Distant metastasis
Stage groups	
Stage 1A	T1; N0; M0; G1
Stage 1B	T2; T3; T4; N0; M0; G1
Stage 2	T1; N0; M0; G2/3
Stage 3A	T2; N0; M0; G2/3
Stage 3B	T3; T4; N0; M0; G2/3
Stage 4	Any T; N1; M0; any G; any T; any N; M1; any G

Malignant retroperitoneal sarcomas are more common than their benign counterparts [8]. Their large sizes often cause compressive symptoms, although they are indolent in nature. Most benign tumors are associated with inherited syndromes, such as von Recklinghausen's disease, and the most common of these tumors is neurogenic in origin, accounting for 30% of benign tumors [9]. Malignant retroperitoneal sarcomas are very rare, accounting for 0.1%-0.2% of all malignancies [8]. There are more than 70 different histological types of soft tissue sarcomas [8]. The most common ones arising from the retroperitoneum are liposarcoma and leiomyosarcoma [5]. Li-Fraumeni syndrome, neurofibromatosis 1, Werner syndrome, and tuberous sclerosis complex are the main hereditary syndromes associated with these tumors [9]. Exposure to radiotherapy as a part of treatment for other malignancies has also been reported as an etiological factor, with the occurrence of these tumors reported within three years of receiving radiotherapy [10].

The imaging investigation of choice is a contrast-enhanced computed tomography of the abdomen [2]. This helps in establishing the location and composition of the tumor and the evidence of necrosis and also detects abdominal metastasis. The CT scan can help in distinguishing retroperitoneal sarcomas from other retroperitoneal tumors such as lymphomas, which often have a multi-nodal involvement, and urogenital tumors such as germ cell tumors, which tend to be more homogenous, the evaluation and treatment of which are often vastly different [2]. Prior to biopsy, a CT scan of the chest is recommended, to rule out pulmonary metastasis [2].

Needle biopsy is warranted to identify the histological type and grading, as the prognosis with each type of retroperitoneal sarcoma and their cure rate with R0/R1 resection are different. CT-guided biopsy through the retroperitoneum is the preferred method for obtaining a histological diagnosis [11]. The collection of six cores with a needle of >16 gauge is recommended. The concerns of tumor dissemination in the needle tract are much lower in the posterior approach, with incidence rates as low as 0.3%, with no impact on the overall survival [11-14].

Where local control is possible, combined radical resection with the excision of the adjacent organs to achieve an R0/R1 resection is the goal of surgical treatment [15]. Such surgeries are often quite challenging, and the accompanying multi-visceral resection increases the morbidity of the surgery. It is important that the tumor and the potentially infiltrated adjacent structures are removed en block in a single session [15-17]. We were able to achieve R0 resection in all our patients probably due to the patient selection based on local infiltration on imaging. The greater the percentage of R0 resection, the better the overall survival [15-17].

Adding radiation therapy in the peri-operative period for the purpose of improving survival in patients with retroperitoneal sarcoma was studied by the American Cancer Society and the American College of Surgeons. According to these authors, patients who received radiation therapy before or after surgery had a better survival rate, as compared to patients who were treated with surgery only [18]. This study, however, did not provide details on the type, method, or dose of radiation used [18]. The STudy of preoperative RAdiotherapy plus Surgery versus surgery alone for patients with retroperitoneal Sarcoma (STRASS) trial looked into this by comparing preoperative radiotherapy with surgery and surgery alone and failed to demonstrate any intra-abdominal recurrence-free survival or overall survival advantage in the experimental arm [19]. Further trials are required to establish the role of radiotherapy in the peri-operative setting.

Doxorubicin is the key drug in systemic treatment for retroperitoneal sarcoma [20]. The addition of ifosfamide has been shown to improve progression-free survival but does not have a significant impact on overall survival. The pathological subset of synovial sarcoma showed significant benefit with chemotherapy [20]. Salvage treatment with the aim of prolonging survival was studied with pazopanib, trabectedin, and eribulin. Pazopanib showed a better prognosis, but this study did not include those patients with liposarcoma [21]. Trabectedin improved survival in liposarcoma and leiomyosarcoma compared to conventional chemotherapy with dacarbazine [22]. Eribulin did not improve the progression-free survival but was seen to improve the overall survival when compared to dacarbazine [23]. Trials on checkpoint inhibitors and the use of neoadjuvant chemotherapy are ongoing, and the results are expected to shed light on the judicious use of cytotoxic drugs for improving outcomes in patients with retroperitoneal sarcoma.

The main limitation of this study is the relatively low number of the study population, in spite of data collection lasting for 10 years, though this is mainly due to the rarity of the tumor as previously described.

## Conclusions

Retroperitoneal tumors are very rare intra-abdominal tumors for which surgical excision with a negative margin is the main prognostic factor for survival. Radiation therapy and chemotherapy can play a role in the peri-operative setting with variable results. The careful selection of patients with the help of imaging and a multidisciplinary tumor board, in a resource-constraint setting, can help in attaining good outcomes.
